# A Case of Severe Dengue With Hemophagocytic Lymphohistiocytosis (HLH) Successfully Managed With Tocilizumab and Dexamethasone

**DOI:** 10.7759/cureus.97054

**Published:** 2025-11-17

**Authors:** Supun Wedasingha, Inu Wijerathna, Pasan Herath, Amila Amarasena, Hemal Senanayake

**Affiliations:** 1 Pharmacology, Faculty of Medicine, University of Rajarata, Anuradhapura, LKA; 2 Internal Medicine, Postgraduate Institute of Medicine, University of Colombo, Colombo, LKA; 3 Hematology, Teaching Hospital Anuradhapura, Anuaradhapura, LKA; 4 Medicine, Faculty of Medicine, University of Rajarata, Anuradhapura, LKA

**Keywords:** dengue hemorrhagic fever, haemophagocytic lymphohistiocytosis, immunosuppressive therapy, multiorgan failure, tocilizumab

## Abstract

Hemophagocytic lymphohistiocytosis (HLH) is a rare, life-threatening condition due to an uncontrolled hyperinflammatory response. While HLH can arise from various causes, it is most commonly triggered by infections. Dengue, a febrile viral illness prevalent in the tropics, can lead to HLH in rare cases. Dengue-associated HLH is particularly challenging to diagnose due to its clinical features overlapping with those of severe dengue. Unless immunosuppressive treatment is promptly initiated, the prognosis of dengue-associated HLH remains poor.

A previously healthy 32-year-old female was admitted to a tertiary care hospital in Sri Lanka with high-grade intermittent fever, gastrointestinal symptoms, and severe dehydration. Initial blood investigations and a point-of-care ultrasound scan confirmed dengue hemorrhagic fever. Despite supportive therapy, the patient's condition deteriorated with persistent fever, severe bicytopenia, and liver impairment. She was diagnosed with HLH based on clinical and laboratory findings, including high ferritin levels and hemophagocytosis in the bone marrow. She was treated with intravenous dexamethasone and tocilizumab, leading to significant clinical improvement. Additionally, therapeutic plasma exchange was performed, contributing to recovery from severe liver dysfunction.

This unique case highlights the importance of early recognition and integration of advanced immunosuppressive therapy in managing dengue-associated HLH. To the best of our knowledge, this is the first reported case where tocilizumab was successfully used in dengue-associated HLH, highlighting the potential of interleukin-6 (IL-6) receptor blockers in similar contexts. The coordinated efforts of hematology, gastroenterology, and general medical teams facilitated timely diagnosis and individualized management, demonstrating how interdisciplinary collaboration can enhance outcomes in complex cases.

## Introduction

Dengue is an acute viral illness caused by a flavivirus, an arthropod-borne virus. The World Health Organization (WHO) recognizes dengue as a significant global public health concern, particularly in tropical and subtropical regions [1]. According to the latest WHO criteria, dengue is further classified as dengue with or without warning signs and severe dengue. Dengue hemorrhagic fever is characterized as a clinical syndrome in which plasma leakage is the hallmark feature [2].

Hemophagocytic lymphohistiocytosis (HLH) is a rare, life-threatening disorder of immune regulation that can lead to hypercytokinemia and immune-mediated injury of multiple organs [3]. The common clinical manifestations of HLH include acute unremitting fever, lymphadenopathy, hepatosplenomegaly, and multiorgan failure [4]. Its complex pathogenesis can give rise to a wide array of non-specific symptoms and signs, which hampers early diagnosis [5]. Dengue-associated HLH can lead to very high mortality due to immune-mediated tissue destruction following the cytokine storm. The diagnosis of HLH can be even more challenging in a patient with dengue due to the overlap of clinical features [6].

Immunosuppressive therapy is the mainstay of treatment in these patients, with high-dose corticosteroids like dexamethasone or methylprednisolone commonly employed to suppress the hyperinflammatory response. In severe HLH and steroid-resistant cases, additional therapies such as etoposide have been recommended [7]. Tocilizumab, an interleukin-6 (IL-6) receptor antagonist, has also been suggested as a useful treatment modality in HLH [4]. We report a case of a patient with dengue hemorrhagic fever complicated with multiorgan failure and HLH. To the best of our knowledge, this is the first case of dengue-associated HLH where the patient was successfully managed with a combination of steroids and tocilizumab.

## Case presentation

A 32-year-old female was admitted to a tertiary care hospital in the North Central Province of Sri Lanka with a four-day history of high-grade intermittent fever with chills and rigors. She reported watery, loose stools, vomiting, and abdominal pain. She also complained of a frontal headache with retroorbital pain, but there was no photophobia or phonophobia. She had significant arthralgia and myalgia as well. She did not have any significant respiratory or urinary symptoms. The patient had intermenstrual bleeding, but no other bleeding manifestations. She did not have a history suggestive of any medical comorbidities.

On admission, she was febrile and severely dehydrated; the capillary refill time was more than two seconds, and she had conjunctival pallor. There was no lymphadenopathy. Her pulse rate was 120 beats per minute, and her blood pressure measured 90/60 mmHg, with no evidence of a postural drop. The respiratory rate was 20 per minute, and oxygen saturation on ambient air was 97%. Physical examination of the cardiovascular, respiratory, abdominal, and neurological systems was all unremarkable. On admission, blood investigations revealed positive NS1 antigen (later, both dengue IgM and IgG antibodies also became positive). The investigations are summarized in Table 1.

**Table 1:** Trend of hematological, biochemical, clotting, and other parameters during the hospital stay

	Normal range	D1	D2	D4	D6	D7	D14
Full blood count							
White blood cell count	(4.0-10.0) 1000/uL	2.2	2.71	4.75	10.24	10.19	7.97
Neutrophils %	(40-80)%	80.5	74.6	43.9	71	77.3	70
Lymphocytes %	(20-40)%	14.5	15.1	50.7	20.5	13.5	16.9
Neutrophil count	(2.0-7.0) 1000/uL	1.77	2.02	2.08	7.27	7.87	5.64
Lymphocyte count	(1.0-3.0) 1000/uL	0.32	0.41	2.41	2.1	11.38	1.35
Red cell count	(4.0-5.5) 1000000/uL	3.96	3.63	3.21	3.09	2.74	4.26
Hemoglobin	(11.0-17.0) g/dL	8.7	8.4	6.9	7.2	6.4	8
Hematocrit	(36.0-50.0)%	28.3	26.5	21.6	21.8	20.1	25.9
Platelet count	(150-400)1000/uL	62	21	15	68	74	188
Renal function tests							
Serum creatinine	(60-120) umol/l	103	96	63	54	59.3	65.18
Blood urea	(2.5-6.4) mmol/l						4.3
Sodium	(135-148) mmol/l	127		138	136	141	140
Potassium	(3.6-5.0) mmol/l	3.9		4.3	3.6	3.5	6
Liver function tests							
Aspartate aminotransferase	(10-35) U/L	501		3560	7130	1700	85
Alanine aminotransferase	(10-40) U/L	281		1880	3080	1090	411
PT-INR				2.6	2.316	1.87	1.42
Inflammatory markers							
C-reactive protein							
Special biochemical investigations							
LDH	(225-450) U/L			4012		1864	
Procalcitonin				0.614			
Ferritin	(13-232) ug/L			3000			
Fibrinogen	(220-426) mg/dL			218			
Triglyceride(mg/dL)	<150 mg/dL			179.64			
Dengue NS 1			Positive				
Dengue IgM			Positive				
Dengue IgG			Positive				

The patient was diagnosed with dengue fever, and the febrile phase monitoring was started according to the local guidelines. On day two of admission, there was a drop in pulse pressure to around 20 mmHg, and a point-of-care ultrasound scan revealed plasma leakage with free fluid in the abdomen. The patient was diagnosed with dengue Hemorrhagic fever (DHF). During the next 48 hours, she was managed in the High Dependency Unit with supportive care including standard fluid therapy according to the National Dengue Management guidelines [8].

Despite supportive therapy, the patient continued to experience high fever spikes and remained hemodynamically unstable. Even though the blood pressure remained marginally low, ischemia was considered unlikely, as both her electrocardiograms and troponin levels were normal. On the seventh day of her illness, she developed signs of acute liver failure, as evidenced by the onset of grade 1 hepatic encephalopathy during clinical assessment, a significant rise in liver enzyme levels (AST: 3560 U/L and ALT: 1880 U/L), and coagulopathy with a PT-INR of 2.6. In response, she was started on an intravenous infusion of N-acetylcysteine (NAC) at a rate of 6.25 mg/kg/hour. Subsequently, the patient became dyspneic and her arterial oxygen saturation dropped to 86% on room air. Arterial blood gas (ABG) analysis revealed type 1 respiratory failure with partially compensated respiratory alkalosis (PaO_2_/FiO_2_ = 221). The chest X-ray revealed pulmonary atelectasis. She was subsequently initiated on high-flow oxygen therapy. Due to concerns of severe sepsis, as indicated by a procalcitonin level of 0.614 ng/mL, intravenous meropenem was also commenced.

Even after completion of 48 hours of the critical phase, the red cell count (lowest red cell count: 2.67 × 10^6^/μL) and platelet count (lowest platelet count: 14 × 10^3^/μL) of the patient kept dropping. She had also developed moderate splenomegaly. The blood picture revealed severe thrombocytopenia with giant platelets. The rotational thromboelastography (ROTEM) study showed overall mild hypercoagulability, mild deficiency of vitamin K-dependent clotting factors, low platelets/platelet dysfunction, and normal fibrinogen levels without any significant hyperfibrinolysis. Her serum ferritin level was more than >3000 ng/mL. Fasting triglycerides were 179.6 mg/dL (<150 mg/dL), and fibrinogen level was 2.18 g/L (2.20 -4.26).

**Figure 1:**
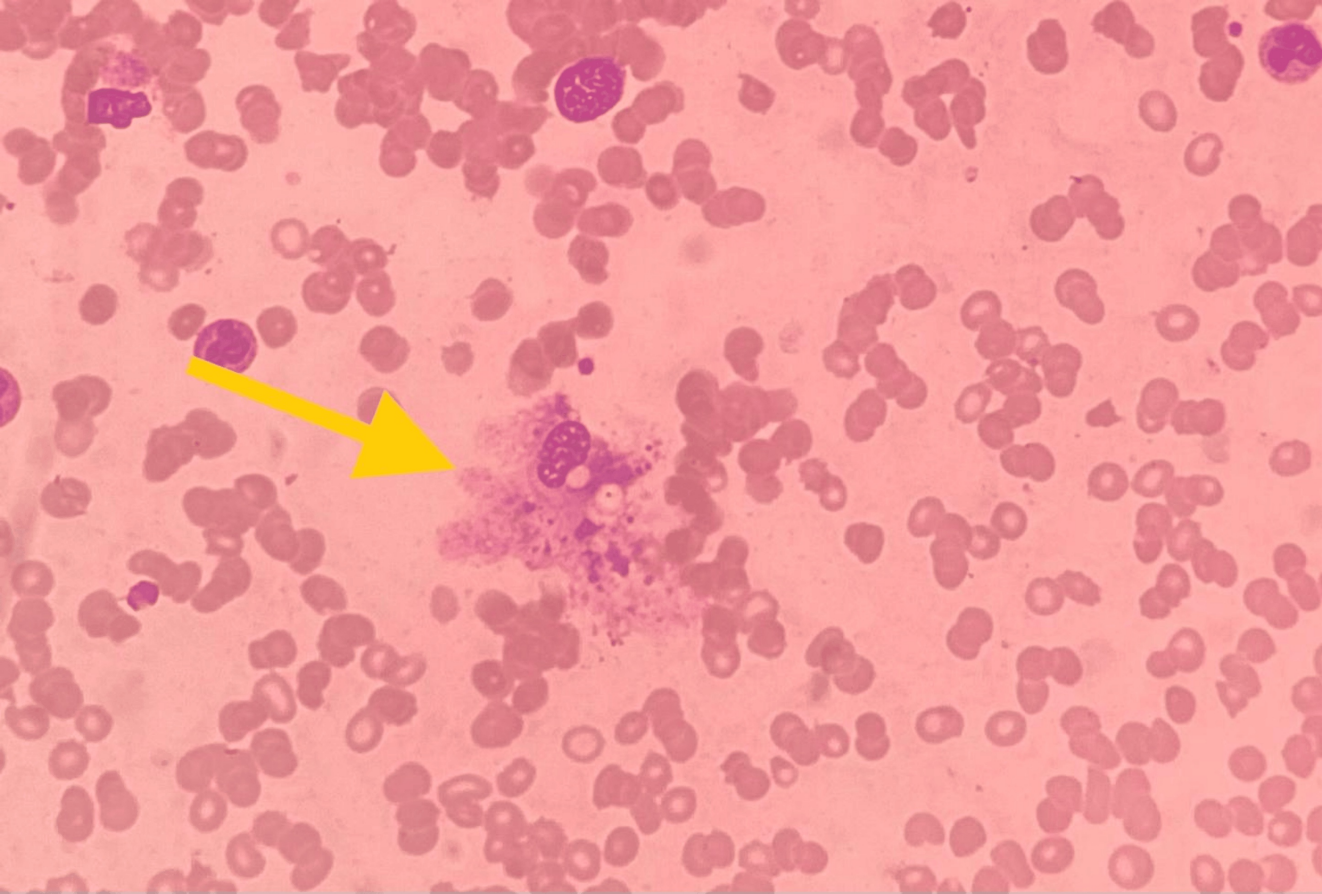
Bone marrow aspirate cytology with Leishman staining showing hemophagocytosis (phagocytosis of red cells and cellular debris)

Bone marrow showed reactive changes, increased osteocytes, and hemophagocytosis (Figure 1). The diagnosis of HLH was confirmed based on HLH-2004 criteria (Table 2). After liaising with the hematology team, the patient was started on intravenous dexamethasone 10 mg per body surface area, and intravenous tocilizumab 8 mg/kg daily was considered since she was clinically deteriorating. Tocilizumab was given for six days, and dexamethasone was continued as a tapering course over the next four weeks.

**Table 2:** Summary of the diagnostic criteria of HLH as per HLH-2004 guidelines*, with corresponding findings in the patient ^*^[9] HLH: hemophagocytic lymphohistiocytosis

	HLH diagnostic criterion (HLH-2004)	Threshold	Patient finding	Is the criterion met?
1	Persistent fever	>7 days	Yes (7+ days of high-grade fever)	Yes
2	Splenomegaly	Clinically or radiologically detected	Yes (moderate splenomegaly)	Yes
3	Cytopenias (at least 2 cell lines affected)			Yes
	Hemoglobin	<9 g/dL	9 g/dL	Yes
	Platelets	<100 × 10³/μL	14 × 10³/μL	Yes
	Neutrophils	<1 × 10³/μL	0.32 × 10³/μL	Yes
4	Hypertriglyceridemia and/or hypofibrinogenemia	Triglycerides ≥265 mg/dL or fibrinogen ≤1.5 g/L	Triglycerides: 153 mg/dL; fibrinogen: 2.18 g/L	No
5	Elevated ferritin	≥500 ng/mL	>3000 ng/mL	Yes
6	Hemophagocytosis in bone marrow, spleen, or lymph nodes	Present	Yes (bone marrow biopsy showed hemophagocytosis)	Yes
7	Low or absent NK-cell activity (where available)	Below the local laboratory reference	Not tested	Not available
8	Elevated soluble IL-2 receptor (sCD25)	≥2400 U/mL	Not tested	Not available

Within 24 hours of starting the immunosuppressive regimen, the patient became fever-free, and platelet count and red cell count started to improve. However, even after 48 hours of IV NAC and 24 hours of immunosuppressive therapy, her liver function continued to deteriorate. Therefore, on day nine of the illness, she underwent one cycle of therapeutic plasma exchange, following which liver derangement showed remarkable improvement. The liver failure regimen was continued up to day 12 of illness. Renal functions remained within the normal range throughout the clinical course. Oxygen therapy was completely discontinued by day 15 of illness as respiratory failure gradually improved with intravenous antibiotics, chest physiotherapy, and incentive spirometry. She was transitioned to oral dexamethasone and was discharged after 14 days of inward management. Her follow-up liver profile and USS abdomen were all normal; previous splenomegaly was not observed. High-resolution CT chest performed one month after the illness was completely normal.

## Discussion

This report describes a previously healthy young female presenting initially with very high-grade fever, arthralgia, myalgia, and dehydration. The detection of plasma leakage on point-of-care ultrasound on the second day of admission was crucial in establishing the diagnosis of DHF. On the seventh day of illness, the patient developed type 1 respiratory failure and severe liver impairment, prompting the initiation of high-flow oxygen therapy and intravenous NAC, respectively. The type 1 respiratory failure was most likely due to pulmonary atelectasis following HLH. Despite continued critical phase monitoring and other supportive treatment modalities, including an acute liver failure regimen, the patient deteriorated clinically with persistent high fever spikes. In addition, she developed bicytopenia and clinically detectable splenomegaly. Her serum ferritin levels were markedly elevated, and subsequent bone marrow examination demonstrated hemophagocytosis.

Thus, the patient met five criteria out of eight criteria, and a diagnosis of HLH could be reliably made. The remarkable recovery of the patient in this case highlights the importance of strict input-output monitoring during the critical phase, supportive therapy whenever necessary, aggressive management of complications, and, most importantly, prompt initiation of immunosuppressive therapy. What makes this case unique is that the patient could be successfully treated with a tapering course of intravenous dexamethasone over four weeks, coupled with six doses of intravenous tocilizumab. Even though tocilizumab has been previously used for HLH, this is the first instance where tocilizumab was successfully incorporated into the treatment of dengue-associated HLH.

Dengue is currently considered one of the most significant neglected tropical diseases [10]. The dengue virus, a member of the genus Flavivirus of the family Flaviviridae, includes four different serotypes (DEN‑1, DEN‑2, DEN‑3, and DEN‑4) [1]. Common clinical features of dengue fever include fever, frontal headache, myalgia, retro-orbital pain, nausea, vomiting, and a blanching macular rash, typically accompanied by leucopenia and varying degrees of thrombocytopenia [11]. Severe dengue is characterized by significant hemorrhage, organ dysfunction, or marked plasma leakage, while all other cases are classified as uncomplicated DF [2]. Diagnosis is typically clinical, based on characteristic symptoms and signs, especially in endemic regions. Confirmation can be obtained through microbiological testing, including virus isolation in cell cultures, nucleic acid detection by PCR, viral antigen detection such as NS1, or serology for specific antibodies. Although no specific antiviral therapy exists for dengue, meticulous attention to fluid management is critical [10]. HLH is a recognized but rare complication of dengue infection [7].

HLH is a rare, life-threatening condition characterized by sustained, but ineffective, immune system activation, resulting in severe and systemic hyperinflammation [5]. Macrophages, NK-cells, and cytotoxic T-lymphocytes are the key cell types involved in the pathogenesis of HLH. Following a trigger, macrophages become activated and secrete cytokines, which in turn can cause organ damage when secreted in excessive amounts [9]. Due to a massive cytokine release, HLH is considered a cytokine storm syndrome [4]. HLH can be primary (due to genetic causes) or secondary to severe infections, malignancy, or autoimmune conditions [9]. Secondary HLH can be triggered by a multitude of infections caused by bacteria, viruses, fungi, and protozoa [4]. According to the criteria introduced by the Histiocyte Society in 2004, a diagnosis of HLH can be reliably made based on the presence of either a molecular diagnosis consistent with HLH and/or five out of eight diagnostic criteria for HLH (HLH-2004 criteria) [9].

Fever, bicytopenia (particularly anemia and thrombocytopenia), hepatomegaly, hepatitis, and elevated ferritin levels (>500 mcg/L) can occur in both severe dengue and HLH. However, compared to severe dengue alone, patients with HLH commonly present with prolonged fever of >7 days, splenomegaly, elevated lactate dehydrogenase, hypertriglyceridemia, hyperfibrinogenemia, and hemophagocytosis on bone marrow examination [12]. Unlike severe dengue, which does not have a specific treatment, dengue-associated HLH can benefit from the prompt initiation of specific therapies targeted at destroying activated immune cells and can suppress the inflammation [12].

Broad aspects of management of secondary HLH include immunosuppressive and immunomodulatory agents, biological response modifiers, and treatment of the underlying condition [9]. Pharmacological therapy is mainly aimed at suppressing the hyperinflammatory state and reversing the immune dysregulation that leads to life-threatening organ dysfunction [8]. Most widely used pharmacological agents in the management of HLH include corticosteroids, intravenous immunoglobulins, etoposide, ciclosporin, and monoclonal antibodies such as alemtuzumab and rituximab [13-15]. Anakinra and tocilizumab are other novel drugs for secondary HLH [16]. Corticosteroids are usually considered the first-line therapy; dexamethasone is preferred over prednisolone as dexamethasone can more readily cross the blood-brain barrier and thus suppress central nervous system inflammation [17]. Most reported cases of dengue-associated HLH have been managed with pulse doses of methylprednisolone or dexamethasone, and intravenous immunoglobulins have also demonstrated effectiveness [18]. In summary, dengue-associated HLH typically responds well to standard HLH treatment protocols [19].

The most critical complication in this case was the development of HLH, confirmed by bone marrow biopsy, and thus fulfilling five out of eight HLH-2004 criteria. Once the diagnosis of HLH was confirmed, the management necessitated a shift from supportive care to aggressive immunosuppressive therapy. The collaboration with the hematology team was crucial in making a prompt diagnosis of HLH and commencing immunosuppressive therapy without delay. Thus, the patient was commenced on intravenous dexamethasone and tocilizumab. Tocilizumab is a monoclonal antibody that inhibits IL-6-mediated signaling by interacting with the soluble and membrane-bound IL-6 receptors (sIL-6R and mIL-6R, respectively) [20]. Tocilizumab was chosen due to its efficacy in modulating the hyperinflammatory response characteristic of HLH. This is because blockade of the downstream signal transduction of IL-6 can prevent the occurrence of cytokine storm and, subsequently, clinical manifestations of HLH [21]. The ultimate recovery of the patient indicated that tocilizumab could be an efficacious treatment in cases of secondary HLH, including dengue-HLH.

Despite aggressive therapy with immunosuppressive agents, the patient's liver functions continued to deteriorate, requiring therapeutic plasma exchange. This intervention once again proved to be crucial, leading to significant improvement in liver functions and overall clinical status. Plasma exchange can be a life-saving intervention in severe dengue with liver failure, thus emphasizing the need for advanced therapeutic options in tertiary care settings [6].

## Conclusions

Features of severe dengue infection can obscure the identification of HLH. Therefore, clinicians should maintain a high index of suspicion for HLH and actively look for the presence of features suggestive of HLH in severe dengue patients who deteriorate despite standard supportive treatment. The successful outcome in this case highlights the significance of adherence to local guidelines as well as international latest updates, the keenness to employ advanced therapeutic interventions when necessary, and the need for a multidisciplinary approach in managing complex cases of dengue fever.
